# Use of Student Evaluation of Teaching (SET) Survey to Evaluate Effectiveness of Teaching in a Leadership Course among Dental Students over Three Years

**DOI:** 10.1155/2020/6436102

**Published:** 2020-06-01

**Authors:** Muhammad Nazir, Asim Al-Ansari, Khalifa AlKhalifa, Balgis Gaffar, Jehan AlHumaid

**Affiliations:** Department of Preventive Dental Sciences, College of Dentistry Imam Abdulrahman Bin Faisal University, P. O. Box 1982, Dammam 31441, Saudi Arabia

## Abstract

Leadership courses are being increasingly integrated into dental curricula. The study aimed to assess the validity and reliability of student evaluation of teaching (SET) instrument among dental students and to evaluate the effectiveness of teaching in a new leadership course over a period of three years. This cross-sectional study was conducted on fourth-year undergraduate dental students (*N* = 260) who took a practice management course over three consecutive years from 2014 to 2016. A 29-item SET questionnaire was administered among students who were willing to participate in the study. Out of 260 students, 185 returned completed surveys and the response rate was 71.15%. Factor analysis (principal component analysis) showed the validity of four dimensions of the SET instrument. Total variance explained by four dimensions was 62.80%. Cronbach's alpha for the instrument was 0.95 and each dimension had fairly high internal consistency (>0.80). Treating students with respect (94%), accepting different viewpoints of students (94.1%), being flexible/open-minded (92.5%), and preparedness in the course (91.9%) were the most common effective teaching traits. Over the period of three years, 16 items showed improvement in teaching and there was a significant improvement in four items (*P* < 0.05). In conclusion, it was found that SET is a valid instrument to evaluate the effectiveness of teaching in nonclinical courses in dentistry. This instrument should be used longitudinally to compare the effectiveness of teaching.

## 1. Introduction

Ongoing technological innovation, greater diversity in treatment options, higher patient demands, and increased health care legislation are bringing challenges for dental professionals [1, 2]. Therefore, effective management of patients, staff, and interaction with colleagues and other health care professionals and stakeholders are becoming more difficult and complex for new dental graduates [3]. Acquiring knowledge and skills in clinical, biomedical, and behavioral sciences in dental programs are not enough to successfully run the business of a dental practice. The understanding and mastery of the principles of business including marketing, human resources, and financial management are needed for graduating dentists in today's highly competitive job market [4]. Hence, the inclusion of a leadership course in dental curricula has been stressed by researchers and practicing dentists to prepare dental students to effectively work as dental practitioners, academicians, and community leaders [5, 6]. A recent study about curricula of practice management courses emphasized the need for improvement in course topics and use of the latest teaching methodologies to enhance the preparedness of graduates for dental practice [7].

The American Dental Association underscores the need for leadership development for dentists and offers several educational opportunities through conferences, podcasts, webinars, and online continuing education activities [8]. The Commission on Dental Accreditation (CODA) has established six standards for dental education programs, and one of the standards is the educational program that includes practice management and health care system [9]. However, leadership competencies are not only required in dental practice management, but also in teaching and learning, research, organized dentistry, community mobilization, and public health [10]. According to the American Dental Education Association Survey of Dental School Seniors (2018), 62.5% of respondents believed that they were prepared in practice administration [11].

In the USA, leadership training is provided by teaching a practice management course in about half of the dental schools; however, various community outreach initiatives and other courses also offer leadership skills in remaining schools [10]. It was reported that dental students expected leadership roles in dental practice (97%) and outreach volunteer opportunities (72%), and the vast majority of them considered leadership competencies important for dental professionals [12]. Literature also showed that about 80% of dental students considered mentorship as the most effective way of learning leadership [13].

A dental practice is the most important place for demonstrating leadership skills, although participating in professional activities and community programs, and taking part in dental associations also provide avenues for dynamic leadership opportunities [14]. Most dental students (84%) from Harvard Dental School of Medicine reported that new leadership practice management course enhanced their interest in communication, leadership, business, team management, dental care access, and practice management [5].

In Saudi Arabia, graduating dental students should be furnished with leadership skills to effectively play their roles in organized dentistry and the provision of dental care particularly to underserved communities. They should also assume a leadership role in their highly competitive and demanding professional careers. The courses, under the name of practice management or administration, vary widely in their content, methodology of delivery, number of credit hours, the academic year of the program, and the level of participation by instructors. For instance, a survey of academic deans about the practice management course showed that 22 practice management courses were taught to third and fourth-year students in 10 Canadian dental schools with 27 to 109 hours of teaching. These courses broadly focused on ethics, human resource management, and private dental practice management [15].

However, focusing on the current and future needs of the dental profession in the Kingdom of Saudi Arabia, a comprehensive course, was developed keeping in mind the national and international course guidelines and standards. A series of in-depth discussions took place among the faculty members of the Dental Public Health Division. The course broadly covers topics on leadership, management of patients and dental team, appointment, inventory, record management systems, health care systems, marketing, and financial management to name a few. Individual and group written assignments, such as case studies, letter writing, SWOT analysis, and team assessment survey, were included in the course. The course also included two guest speaker presentations. Over the period of three years, new topics were included in the course each year based on the input from faculty members, guest speakers, students, and emerging evidence in the practice management. The new onecredit course was delivered to fourth-year students and completed in one semester.

A well-designed practice management course is one of many ways to teach management and leadership knowledge and skills to dental students. The success of a course depends upon the effectiveness of teaching which is usually evaluated by students, peers, and the faculty, but student evaluation is the most commonly done at the level of university [16]. However, dental students' evaluation of teaching (SET) through a validated survey is scant in the literature. Therefore, the study aimed to evaluate (1) the validity and reliability of the SET instrument by performing its psychometric analysis and (2) the effectiveness of teaching in a leadership course over a period of three years.

## 2. Materials and Methods

### 2.1. Study Design and Participants

This cross-sectional study was conducted on undergraduate fourth-year dental students at the College of Dentistry, Imam Abdulrahman Bin Faisal University, Dammam, Saudi Arabia. The college is an accredited public dental institute in the Eastern province of the country. The Bachelor of Dental Surgery (BDS) program includes students from the second year to six year. However, the first year of these students is spent in learning science subjects at the Preparatory College in the University. The study analyzed the data of fourth-year students who participated in practice management, nonclinical, course over a period of three years. In 2014, the course was introduced to students (*N* = 72) who provided their responses to students' evaluations of teaching (SET) instrument at the end of the course. Next year, another group of fourth-year students (*N* = 94) attended the same course and their data were collected using the same instrument. The third batch of the fourth-year students (*N* = 94) took the course in 2016 and their responses were gathered at the end of the course using the same instrument. Therefore, the data of fourth-year students from three batches of consecutive students were collected for the study. A convenience sample was used for the study by inviting all the students in three batches of fourth-year class over three years (*N* = 72 + 94 + 94 = 260 students).

### 2.2. Data Collection Instrument

The students' evaluations of teaching (SET) instruments are used to evaluate the effectiveness of teaching in higher education. These instruments are considered more important than peer evaluation and faculty self-reports [16]. Dodeen validated a SET survey for the students of humanities, sciences, business, IT, agriculture, engineering, and law colleges in the Middle East. The instrument is not subject matter specific and is neither too short nor too long [16]. Therefore, the current study administered the same SET survey among dental students in Saudi Arabia [16]. The questionnaire comprised 29 items distributed in five dimensions such as teachers' knowledge and organization, clear explanation, grading and evaluation, teaching methods, and relationship with students. There are 7 items in the “Teachers' Knowledge and Organization” domain, 6 items in the “Clear Explanation” domain, 6 items in the “Grading and Evaluation” domain, 4 items in the “Teaching Methods” domain, and 6 items in “Relationship with Students” domain. A 5-point Likert scale (from very poor = 1 to very good = 5) was used for each item of the instrument [16].

### 2.3. Procedures

A self-administered questionnaire in English was distributed among dental students. English is the medium of instruction in the College. Each year, the Vice Dean of Academic Affairs granted permission to distribute questionnaires. The copies of questionnaires were provided to all students in the classes at the end of the course during the last weeks of the first semester each year. One researcher briefed students about the survey, and they were encouraged to ask questions if any item needed further explanation. They were informed about no negative consequences in case students refusing to participate in the study. The confidentiality and privacy of students' responses were maintained by administering an anonymous questionnaire. Those students who agreed to voluntary participation were included in the survey. The questionnaire administration was completed by one researcher within approximately 20 minutes. Forty-nine students in 2014, 72 students in 2015, and 64 students in 2016 returned completed questionnaires. Of 260 students who took the course over three years, 185 filled questionnaires, and the response rate was 71.15%.

### 2.4. Statistical Analysis

The data were entered in MS Excel (2010) and transferred to IBM SPSS Statistics for Windows, version 22 (IBM Corp., Armonk, N.Y., USA) for statistical analysis. Descriptive statistics included means, standard deviations, and frequency distributions. The psychometric analysis of the questionnaire was performed to evaluate its validity and reliability. Exploratory Factor Analysis (EFA) was performed to determine the construct validity of the questionnaire [17, 18]. The reliability of the questionnaire can be assessed by the internal consistency of the instrument [18]. The internal reliability of the instrument is measured by Cronbach's alpha and 0.7 is its minimum recommended value. Internal reliability of each scale, as well as the correlation of items in the questionnaire, were computed [19]. Independent sample *t*-test was performed to report differences in the mean score of responses by male and female students. Kruskal-Wallis Test was performed to evaluate the effectiveness of teaching over three years. For a better interpretation of data, very poor and poor response were combined to present poor, and good and very good responses were combined to present good as mentioned above. A *P* value of 0.05 was used for statistical significance.

## 3. Results

The validity of the instrument was confirmed by the factor analysis shown in Table 1. Kaiser's criterion of Eigen values ≥ 1 and scree plot were used for factor extraction. Total variance explained by four scales was 62.80%. Preparation and assessment (Factor 1) accounted for 20.71% of the variance and interaction with students (Factor 2) represented 15.64% of the variance, while teaching and learning (Factor 3) and preparedness of instructor (Factor 4) explained 14% and 12.44% of the variance, respectively (Table 1). High internal consistency of the instrument and each scale confirmed the reliability of the instrument. Cronbach's alpha for the instrument was 0.95 and each dimension had fairly high internal consistency (Cronbach's for preparation and assessment = 0.93, interaction with students = 0.87, teaching and learning = 0.89, and preparedness of instructor = 0.80).

Table 2 shows students' responses about the effectiveness of teaching during the last three years. It can be seen that most students reported that treating students with respect (94%), accepting different viewpoints presented by students (94.1%), being flexible/open-minded when dealing with students (92.5%), and preparedness (91.9%) in the course were good or very good. No statistically significant differences were observed between male and female students' responses.

The comparison of students' responses over three years showed that there was improvement in more than half of the items of effective teaching however, statistically significant improvement was seen in four items (*P* < 0.05) (Table 3).

## 4. Discussion

The SET instrument is a multidimensional, reliable, stable, and valid tool and is used frequently to obtain diagnostic feedback on teaching by instructors. Other purposes of collecting SETs include course selection by students and decision-making about staff's teaching effectiveness by administrators [20]. Our study evaluated the psychometric properties of a SET instrument among dental students in a leadership, nonclinical, course. This revalidation of the instrument has provided valuable information and it can be used to obtain a comprehensive and multidimensional assessment of the effectiveness of teaching in nonclinical undergraduate courses in dentistry. In our study, construct validity was confirmed by the factor analysis that grouped items into four dimensions/scales and the instrument showed high internal consistency (Cronbach's alpha 0.95).

Revalidation of the SET instrument should be based on a theoretical model in teaching or empirical testing that involves procedures to ensure its validity and reliability [21]. The present study evaluated the validity of the instrument by performing factor analysis. The original instrument from the previous study consisted of 29 items in five dimensions [16]. In contrast, our analysis of data organized the items into four dimensions containing varying number of items in each dimension compared to the previous study [16]. However, similar to our findings, Gurosy and Umbreit reported four dimensions of the SET instruments [22]. It is also known that most SET instruments broadly contain three dimensions, namely, organization, student grading, and instructor-student interaction [16]. These discrepancies in the content and construction of different SET instruments could be related to varying expectations and needs of various institutions [23]. Moreover, these variations could have resulted because of the number and types of participants and methodological differences including data analysis techniques.

When students' responses about different dimensions of the instrument were analyzed then instructor-student relationship and instructor's preparedness (organization) stood out as ≥90% of students claimed that instructors treated students with respect, accepted different viewpoints, demonstrated flexibility, and were prepared in the course. Schonwetter et al. [24] found that 30% (*n* = 102) of students in classroom teaching reported the organization and 27% (*n* = 91) interaction of teacher with students/individual rapport with students were the most common characteristics of effective teachers while only 4% considered assessment an element of effectiveness of teaching. Similarly, Jahangiri and Mucciolo identified development, organization, and design of content (>50%) and interaction with students (34.6%) and caring attitudes (28.8%) as the most predominant features of teacher's effectiveness in nonclinical programs [25]. In a study by McAndrew et al. students reported that student-teacher interaction/character (50%) was the most common theme of characteristics of effective teachers, followed by competence/preparedness of instructor theme (34%) and communication (17%) [26].

 Student evaluation of teaching is widely used to assess the effectiveness of teaching in higher education globally. It is also documented that SETs can be used to obtain students' feedback to monitor the quality of teaching to serve the purpose of quality assurance management in universities [27]. In addition, it shows the promise of the institution to monitor teaching performance as part of continuous improvement activities in the program [28]. It has been suggested that meaningful inferences can be drawn from the results of evaluations that are conducted repeatedly at regular intervals to ensure the improvement process in teaching. Moreover, these evaluations should be combined with the testing of data [29].

The improvement in teaching observed in successive years was reported in 8 out of 14 studies involving longitudinal comparisons of students' evaluations [30]. In the present study, student feedback was observed longitudinally over a period of three years to report the quality of teaching provided in the course. Results obtained through the analysis of data showed that 16 of 29 items of the SET instrument demonstrated improvement in teaching from 2014 to 2016. Overall, the mean score of these 16 items increased, but significant increase was observed in four items: “encourage students to seek knowledge from multiple resources” (*P*=0.024), “offer useful feedback on tests” (*P*=0.001), and “emphasize difficult points and facts” (*P*=0.036). On the other hand, the mean score in two items such as “examination questions are clear” (*P*=0.003) and “flexible/open-minded when dealing with students” (*P*=0.021) significantly decreased thus requiring improvement in these areas. These results support the common notion that improvement in teaching can be obtained by using student evaluations.

There are claims about the positive and valuable influence of student evaluation of teaching on academic standards [30]. There are also concerns and confusion about student evaluation. It has been argued the student rating of effective teaching can provide inaccurate inferences leading to unfair decision including enhancing student grades and negatively affecting standards of teaching [30, 31]. However, the literature lacks convincing evidence about the impact of student evaluation on inflating student grades, lowering course requirement, and compromising teaching standards [30].

As discussed previously, student evaluation of teaching has benefits of improving the effectiveness of teaching, academic standards, and quality assurance management. It is, therefore, suggested that student evaluations should be combined with self-evaluation and peer assessment because student rating alone is an inadequate measure of teaching effectiveness as students have limitations of assessing the appropriateness of course material and assessment methods [31–33]. Therefore, in the present study, student evaluations in addition to the feedback of faculty members and guest speakers and self-assessment were used to improve the quality of the course by modifying its content, delivery, and assessment.

Nonparticipation (28.85%) in the present study was mainly because of the absence of some students on the day of questionnaire administration. The college offers an intense undergraduate dentistry program; hence some students tend to avoid attending the last session of certain courses so that they can better prepare for their final examinations. A large data set obtained from a bigger sample of students could have better captured the study outcomes. Regardless of this limitation, this study provided an opportunity to guide best practices in teaching nonclinical courses in dentistry.

## 5. Conclusions

Based on the study findings, it is concluded that SET, a valid and reliable instrument, can be used to evaluate the effectiveness of teaching in nonclinical courses in dentistry. Longitudinal comparison of student's evaluations over the years can contribute to improving teaching. It is emphasized that a validated SET instrument should be used as part of evidence-based practices in dental education to evaluate the effectiveness of teaching. Moreover, data should be monitored longitudinally over consecutive years particularly to identify areas needing improvement and to maintain elements of effective teaching in the course. Dental institutions, educational policymakers, and instructors should ensure that the course content, teaching methods, assessment instruments are continuously updated using student evaluation in conjunction with self-evaluation and peers' observations.

## Figures and Tables

**Table 1:** Factor analysis of students' evaluations of teaching.

	Factor 1 preparation and assessment	Factor 2 interaction with students	Factor 3 teaching and learning	Factor 4 preparedness of instructor
Clear presentations of course materials	0.757			
Assignments, projects, activities, ... etc. are clear	0.656			
Examples are used to simplify difficult points	0.667			
Clear explanations of concepts and principles	0.657			
Emphasize difficult points and facts	0.659			
Examination questions are clear	0.472			
Examination covers content emphasized by the instructor	0.523			
Grading criteria are clear	0.403			
Offer useful feedback on assignments, projects, activities	0.575			
Offer useful feedback on tests	0.678			
Instructor's grading policy is fair	0.585			
Use variety of assessment methods	0.534			
Available outside the classroom for assisting students	0.506			
Cares for students' learning		0.663		
Treat students with respect		0.719		
Accept different viewpoints presented by students		0.733		
Treat all students fairly		0.728		
Flexible/open-minded when dealing with students		0.708		
Effective use of class time			0.679	
The class time is carefully planned			0.761	
Effective classroom management			0.574	
Use teaching aids and technology effectively			0.597	
Present course materials at an appropriately paced sequence			0.535	
Encourage students to seek knowledge from multiple resources			0.647	
Motivate students to learn			0.628	
The instructors are well-prepared in their course				0.593
The instructors are informative when responding to students' questions				0.631
The instructors state goals and objectives clearly				0.518
Lectures are well organized				0.615
Eigenvalue	13.76	1.92	1.36	1.15
% of total variance	20.71	15.64	14	12.44
Total variance	62.80			

**Table 2:** Students' responses about the effectiveness of teaching.

Assessments and evaluation	Poor (%)	Moderate (%)	Good (%)
Examination questions are clear	4.9	21.1	74.1
Examination covers content emphasized by the instructor	1	18.4	80.5
Grading criteria are clear	3.7	15.7	80.6
Clear presentations of course materials	4.3	20.0	75.7
Assignments, projects, activities, ...etc. are clear	10.3	34.6	55.1
Examples are used to simplify difficult points	6.5	19.5	74.1
Clear explanations of concepts and principles	3.8	24.9	71.4
Emphasize difficult points and facts	5.9	30.3	63.7
Offer useful feedback on assignments, projects, activities	11.4	25.4	63.2
Offer useful feedback on tests	6	18.9	75.2
Instructor's grading policy is fair	2.7	13.0	84.3
Use variety of assessment methods	1.6	18.9	79.5
Available outside the classroom for assisting students	2.1	13.5	84.3

*Interaction with students*			
Care for students' learning	2.1	9.2	88.7
Treat students with respect	2.2	3.8	94
Accept different viewpoints presented by students	1	4.9	94.1
Treat all students fairly	4.9	10.8	84.3
Flexible/open-minded when dealing with students	1.6	5.9	92.5

*Teaching and learning*			
Effective use of class time	15.1	36.2	48.7
The class time is carefully planned	12.9	34.6	52.4
Effective classroom management	11.9	24.9	63.2
Use teaching aids and technology effectively	8.1	31.4	60.5
Present course materials at an appropriately paced sequence	3.3	23.2	73.5
Encourage students to seek knowledge from multiple resources	5.4	24.3	70.3
Motivate students to learn	5.4	22.7	71.9

*Preparedness of instructor*			
The instructors are well-prepared in their course	0.5	7.6	91.9
The instructors are informative when responding to students' questions	2.2	8.6	89.2
The instructors state goals and objectives clearly	2.2	11.4	86.5
Lectures are well organized	2.7	13.5	83.8

**Table 3:** Students' responses about the effectiveness of teaching over last three years.

Assessments and evaluation	Year 2014/15	Year 2015/16	Year 2016/17	*P* value
	Mean	Mean	Mean	
Examination questions are clear	4.04	4.01	3.81	0.003^*∗*^
Examination covers content emphasized by the instructor	4.24	4.14	4.00	0.143
Grading criteria are clear	4.27	4.11	4.21	0.632
Clear presentations of course materials	4.04	3.98	4.23	0.718
Assignments, projects, activities, ... etc. are clear	3.84	3.47	3.81	0.105
Examples are used to simplify difficult points	3.94	3.88	4.23	0.107
Clear explanations of concepts and principles	3.98	3.88	4.14	0.897
Emphasize difficult points and facts	3.61	3.81	4.14	0.036^*∗*^
Offer useful feedback on assignments, projects, activities	3.76	3.66	4.02	0.195
Offer useful feedback on tests	3.53	4.22	4.16	0.001^*∗*^
Instructor's grading policy is fair	4.12	4.26	4.26	0.511
Use variety of assessment methods	3.88	4.18	4.19	0.044^*∗*^
Available outside the classroom for assisting students	4.14	4.26	4.40	0.118

*Interaction with students*				
Care for students' learning	4.33	4.30	4.49	0.231
Treat students with respect	4.59	4.47	4.72	0.546
Accept different viewpoints presented by students	4.49	4.48	4.51	0.879
Treat all students fairly	4.55	4.17	4.33	0.166
Flexible/open-minded when dealing with students	4.69	4.39	4.56	0.021^*∗*^

*Teaching and learning*				
Effective use of class time	3.51	3.40	3.84	0.950
The class time is carefully planned	3.67	3.48	3.67	0.366
Effective classroom management	3.86	3.63	3.95	0.315
Use teaching aids and technology effectively	3.59	3.72	3.91	0.445
Present course materials at an appropriately paced sequence	3.92	3.88	4.12	0.268
Encourage students to seek knowledge from multiple resources	3.94	3.82	4.23	0.024^*∗*^
Motivate students to learn	4.04	3.88	4.23	0.749

*Preparedness of instructor*				
The instructors are well-prepared in their course	4.53	4.38	4.49	0.350
The instructors are informative when responding to students' questions	4.51	4.27	4.44	0.405
The instructors state goals and objectives clearly	4.31	4.20	4.44	0.411
Lectures are well organized	4.14	4.17	4.23	0.601

## Data Availability

The SPSS data file of this study is available from the corresponding author upon request.
